# Association between XRCC1 polymorphisms and the risk of cervical cancer: a meta-analysis based on 4895 subjects

**DOI:** 10.18632/oncotarget.13663

**Published:** 2016-11-26

**Authors:** Xianling Zeng, Yafei Zhang, Ting Yue, Taohong Zhang, Junxia Wang, Yan Xue, Ruifang An

**Affiliations:** ^1^ Department of Obstetrics and Gynecology, the First Affiliated Hospital of Xi’an Jiaotong University, Xi’an Shaanxi 710061, China; ^2^ Department of General Surgery, the Second Affiliated Hospital of Xi’an Jiaotong University, Xi’an Shaanxi 710004, China

**Keywords:** X-ray repair cross complementing 1, polymorphism, cervical cancer, meta-analysis

## Abstract

The present meta-analysis was intended to explore the relationship between the X-ray repair cross complementing 1 (XRCC1) polymorphisms (Arg194Trp, Arg280His and Arg399Gln) and cervical cancer risk. Several electronic databases were searched systematically and bibliographies of relevant papers were identified carefully. Then, a meta-analysis was performed based on eligible studies in various genetic models. Pooled odds ratios (OR) with 95% confidence intervals (95% CI) were employed to evaluate the strength of associations between the XRCC1 polymorphisms and cervical cancer risk. Additionally, heterogeneity analysis and sensitivity analysis were done if necessary. Totally, 11 articles involving 2092 cases and 2803 controls were included. Taken together, there was no obvious association between the Arg194Trp or Arg280His polymorphism and cervical cancer risk. Considering the great heterogeneity, subgroup analysis was done, but the pooled result remained stable. Nevertheless, the association between the Arg399Gln polymorphism and cervical cancer risk showed distinct statistic significance in the allele model, dominant model, homozygous model and heterozygous model. In view of the exiting heterogeneity, we did subgroup analysis stratified by ethnicity, resulting in the fact that the Arg399Gln polymorphism was related to the decreased risk of cervical cancer. The Begg's test and Egger's test were used to find no publication bias. To conclude, the current meta-analysis indicated that the XRCC1 Arg399Gln polymorphism decreased the risk of cervical cancer, while the Arg194Trp and Arg280His polymorphisms were not associated with cervical caner risk. Certainly, a well-designed large-scale multicenter study is warranted to confirm the finding.

## INTRODUCTION

Nowsdays, cervical cancer is one of the most common genital tract carcinomas and has become a challenging health issue confronted by women throughout the world. It seriously threatens women's quality of life arising from reproductive endocrine function's damage caused by this malignancy, and brings about great morbidity and economic burden. Infection with high-risk types of human papillomavirus (HPV) is the main causative factor for developing cervical intraepithelial neoplasia (CIN) which is a precursor lesion for cervical cancer. While, not all women who are infected with HPV will certainly progress into cervical cancer, suggesting that there are still other factors playing a role in the pathogenesis of cervical cancer. For example, ultraviolet, ionizing radiation and environmental chemical agents can lead to DNA damage, initiating certain human cancers [1–9].

In human body, DNA repair genes are considerable factors in the prevention of genomic injury and sequential carcinogenesis. So variants of DNA repair genes might be able to impair DNA repair ability and have been suggested to be associated with cancer risk. X-ray repair cross complementing group 1 ( XRCC1) gene is a typical DNA repair gene. It is located at chromosome 19q13.2-13.3 and encodes the scaffolding protein [10]. The protein functions in the repair of single-strand breaks which is the most common lesions in cellular DNA [11]. Both biological and biochemical evidence indicate XRCC1 interacts with a complex of DNA repair proteins, such as poly(ADP-ribose) polymerase [11–13]. There are three most common polymorphisms in XRCC1, contributing to amino acid substitutions in XRCC1 at codon 194 (exon 6, base C to T, amino acid Arg to Trp), codon 280 (exon 9, base G to A, amino acid Arg to His), and codon 399 (exon 10, base G to A, amino acid Arg to Gln) (http://egp.gs.washington.edu). And eventually these variants alter XRCC1 function.

A great many epidemiologic studies have been conducted to evaluate the role of the XRCC1 polymorphisms (Arg194Trp, Arg280His and Arg399Gln) on cervical cancer risk [14–24]. But the results were inconclusive. For example, Zhang et al. found the XRCC1 Arg194Trp polymorphism showed no significant association with CIN and squamous cervical carcinomas (SCC), while the Arg280His polymorphism acted as a protective factor for SCC, and the Arg399Gln polymorphism increased CIN risk among women who first gave birth before 22 years old [14]. Bajpai et al. suggested XRCC1 polymorphisms (Arg194Trp, Arg280His and Arg399Gln) increased cervical cancer risk greatly [23]. Barbisan et al. convinced that XRCC1 polymorphisms (Arg194Trp and Arg399Gln) genotypes and haplotypes contributed to reducing the risk of cervical cancer development in Argentin women [22]. Facing the contradictory, we assumed that a meta-analysis of various studies involving more subjects would offer a more precise conclusion. Thus, we aimed to obtain the summary risk estimating the association between the above mentioned three polymorphisms of XRCC1 and cervical cancer risk through a meta-analysis.

## RESULTS

### Characteristics of included studies

**Figure 1:**
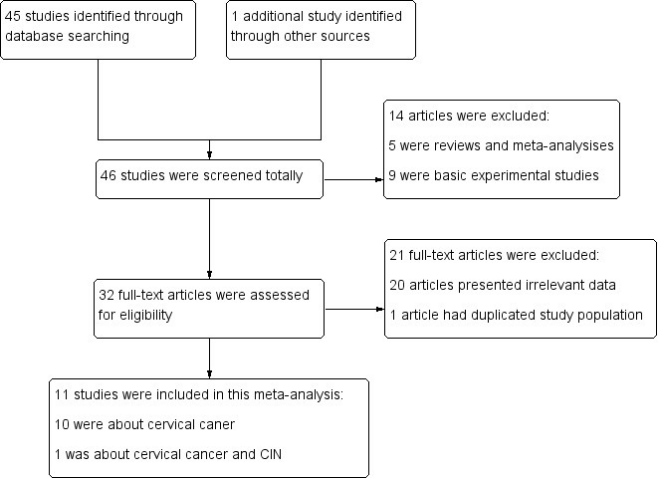
Search flow diagram

We initially retrieved 46 articles through various electronic databases. After removing reviews, meta-analysises, basic experimental studies, we got 32 articles needing screening the full-text. While 20 articles did not present available data and one was a duplicate study [25]. Consequently, a total of 11 articles involving 2092 cases and 2803 controls were recruited in the present meta-analysis [14–24] (Figure 1). Among these articles, 7 articles were about Arg194Trp (rs1799782) [14, 16, 17, 20–23], 4 articles were about Arg280His (rs25489) [14, 15, 20, 23], and 11 articles were about Arg399Gln (rs25487) [14–24]. However, we only recruited 10 studies when analyzing the association between Arg399Gln polymorphism and cervical cancer risk because an article offered data concerning CIN and cervical cancer as a whole [16]. Yet the study was included in the subgroup analysis stratified by the degree of cervical lesions.

The included studies were all performed in recent years. The objects in eight studies were of Caucasians, two were of Asian and one was Mixed. Eight out of eleven control groups were population-based or healthy-based participants and the ramaining three were hospital-based. The largest number of subjects was 1339, almost 10-fold of the smallest number (*n* = 133). (Table 1) The quality assessment of included studies showed that all the studies were of high quality except that one study scored 5 points. (Table 2)

**Table 1:** Characteristics of the studies included in the meta-analysis

First author	Year	Country	Ethnicity	Source of controls	Genotyping method
Alsbeih et al. [24]	2013	Saudi Arabia	Asian	Hospital-based	PCR
Bajpai et al. [23]	2016	India	Asian	Hospital-based	PCR-RFLP
Barbisan et al. [22]	2011	Argentina	Caucasian	Population-basd	PCR
Djansugurova et al. [21]	2013	Kazakhstan	Asian	Healthy-based	PCR
Huang et al. [20]	2007	China	Asian	Population-basd	MA-PCR
Rozak et al. [18]	2011	Poland	Caucasian	Hospital-based	PCR-RFLP
Niwa et al. [19]	2005	Japan	Asian	Healthy-based	PCR
Setthetham-Ishida et al. [17]	2011	Thailand	Asian	Healthy-based	PCR-RFLP
Wang et al. [16]	2009	Costa Rica	Mixed	Population-basd	Taqman
Wu et al. [15]	2004	Taiwan	Asian	Population-basd	PCR-RFLP
Zhang et al. [14]	2012	China	Asian	Healthy-based	PCR

PCR: polymerase chain reaction; RFLP: restriction fragment length polymorphism.

**Table 2:** Quality assessment of studies based on the modified scoring system [31]

Study name	Representativeness of cases	Source of controls	HWE in controls	Genotyping examination blinded	Association assessment	Total
Alsbeih 2013	2	1	1	0	1	5
Bajpai 2016	2	1	2	0	2	7
Barbisan 2011	1	2	2	0	1	6
Djansugurova 2013	2	2	2	0	1	7
Huang 2007	2	2	2	0	2	8
Rozak 2011	2	1	2	0	1	6
Niwa 2005	2	2	2	0	1	7
Setthetham-Ishida 2011	2	2	2	0	2	8
Wang 2009	2	2	2	0	1	7
Wu 2004	1	2	2	0	2	7
Zhang 2012	2	2	2	0	2	8

HWE : Hardy-Weinberg equilibrium.

Hardy-Weinberg equilibrium (HWE) examination results of the included studies and the XRCC1 polymorphisms genotype distribution in cases and controls were displayed in Table 3. All studies were consistent with HWE except for three studies for Arg194Trp [17, 21, 22], one study for Arg280His [23], and one study for Arg 399Gln [24].

**Table 3:** XRCC1 polymorphisms genotype distribution and allele frequency in cases and controls

First author	Genotype (*N*)								Allele frequency (*N*)				HWE
	Case				Control				Case		Control		
**Arg194Trp(rs1799782)**	Total	TrpTrp	ArgTrp	ArgArg	Total	TrpTrp	ArgTrp	ArgArg	Trp	Arg	Trp	Arg	
Bajpai et al. [23]	65	38	16	11	68	13	11	44	92	38	37	99	1.07
Barbisan et al. [22]	103	4	20	79	114	4	12	98	28	178	20	208	< 0.05
Djansugurova et al. [21]	217	6	48	163	160	15	40	105	60	374	70	250	< 0.05
Huang et al. [20]	539	78	220	241	800	63	330	407	376	702	456	1144	0.73
Setthetham-Ishida et al. [17]	111	9	49	53	118	2	51	65	67	155	55	181	0.02
Wu et al. [15]	100	9	43	48	196	16	93	87	61	139	125	267	0.20
Zhang et al. [14]	80	8	31	41	177	19	71	87	47	113	109	245	0.43
**Arg280His (rs25489)**	Total	HisHis	ArgHis	ArgArg	Total	HisHis	ArgHis	ArgArg	His	Arg	His	Arg	
Bajpai et al. [23]	65	39	6	20	58	3	7	48	84	46	13	103	< 0.05
Huang et al. [20]	539	6	117	416	800	9	171	620	129	949	189	1411	0.46
Wu et al. [15]	100	2	24	74	196	1	55	140	28	172	57	335	0.07
Zhang et al. [14]	80	1	11	68	177	1	34	142	13	147	36	318	0.49
**Arg399Gln ( rs25487)**	Total	GlnGln	ArgGln	ArgArg	Total	GlnGln	ArgGln	ArgArg	Gln	Arg	Gln	Arg	
Alsbeih et al. [24]	100	14	34	52	100	1	40	59	62	62	158	42	0.04
Bajpai et al. [23]	65	31	22	12	68	12	33	23	84	84	79	57	0.989
Barbisan et al. [22]	103	18	31	54	114	18	59	37	67	67	133	95	0.49
Djansugurova et al. [21]	217	20	119	78	160	4	90	66	159	159	222	98	4.21
Huang et al. [20]	539	47	203	289	800	37	235	528	297	297	1291	309	0.10
Rozak et al. [18]	189	39	101	49	308	40	152	116	179	179	384	232	0.37
Niwa et al. [19]	131	13	49	69	320	26	109	185	75	75	479	161	0.097
Setthetham-Ishida et al. [17]	111	4	41	66	118	5	44	69	49	49	182	54	0.54
Wu et al. [15]	100	8	38	54	196	9	73	114	54	54	301	91	0.53
Zhang et al. [14]	80	6	31	43	177	10	58	109	43	43	276	78	0.54

HWE: Hardy-Weinberg equilibrium.

### Meta-analysis results

For XRCC1 Arg194Trp polymorphism, there were seven studies, involving 1315 cases and 1633 controls, evaluating the connection between it and cervical cancer susceptibility. All the studies were done among the Asian population apart from one study [22]. Overall, there was no obvious statistic significance between the polymorphism and cervical cancer in all five models (*P* > 0.05). considering the moderate to great heterogeneity among studies, we performed subgroup analysis stratified by the degree of cervical lesion. However, the finding that the pooled OR still incorporated 1.0 showed that the Arg194Trp polymorphism had no association with the risk of cervical cancer. Then we excluded three studies which were not consistent with HWE [17, 22, 23] and reassessed the relationship between this locus and cervical cancer risk. The final results did not change substantially. (Table 4).

**Table 4:** Meta-analysis results

Subgroup Analysis		OR	95% CI	*P* value	Heterogeneity		Effects model
					*I*^2^	*P* value	
**XRCC1 Arg194Trp**		**Allele model (Trp vs. Arg)**					
Overall		1.04	0.80–1.36	0.75	72%	0.001	R
Degree of cervical lesion	CC	1.03	0.65–1.62	0.91	78%	0.004	R
	CC+CIN	1.66	0.83–3.31	0.15	94%	< 0.00001	R
Dominant model (TrpTrp + ArgTrp vs. ArgArg)							
Overall		1.12	0.96–1.31	0.61	49%	0.07	F
Degree of cervical lesion	CC	1.03	0.66–1.61	0.89	65%	0.04	R
	CC+CIN	1.80	0.77–4.21	0.18	92%	< 0.00001	R
Recessive model (CC vs. GC + GG)							
Overall		1.08	0.60–1.94	0.81	72%	0.002	R
Degree of cervical lesion	CC	1.03	0.35–3.10	0.95	72%	0.01	R
	CC+CIN	1.76	0.95–3.24	0.07	71%	0.03	R
Homozygous genetic model (TrpTrp vs. ArgArg)							
Overall		1.13	0.61–2.12	0.69	71%	0.002	R
Degree of cervical lesion	CC	0.54	0.40–0.74	0.95	14%	0.004	R
	CC+CIN	0.75	0.57–0.97	0.12	87%	0.0005	R
Heterozygous genetic model (ArgTrp vs. ArgArg)							
Overall		1.07	0.91–1.26	0.43	10%	0.35	F
Degree of cervical lesion	CC	0.56	0.41–0.77	0.84	43%	0.15	R
	CC+CIN	0.89	0.67–1.16	0.23	87%	0.0004	R
**XRCC1 Arg280His**		Allele model (His vs. Arg)					
Overall		1.78	0.63–5.01	0.28	95%	< 0.00001	R
Dominant model (HisHis + ArgHis vs. ArgArg)							
Overall		1.16	0.94–1.43	0.17	90%	< 0.00001	R
Recessive model (HisHis vs. ArgHis + ArgArg)							
Overall		4.08	0.58–28.75	0.16	82%	0.0009	R
Homozygous genetic model (HisHis vs. ArgArg)							
Overall		4.12	0.55–30.79	0.17	83%	0.0006	R
Heterozygote genetic model (ArgHis vs. ArgArg)							
Overall		0.97	0.77–1.21	0.78	0%	0.41	F
**XRCC1 Arg399Gln**		Allele model (Gln vs. Arg)					
Overall		0.39	0.29–0.51	< 0.00001	83%	< 0.00001	R
Ethnicity	Asian	0.34	0.26–0.43	0.00001	72%	0.0008	R
	Caucasian	0.63	0.51–0.79	< 0.0001	0%	0.51	R
Degree of cervical lesion	CC	0.41	0.31–0.54	< 0.00001	72%	0.001	R
	CC+CIN	0.39	0.24–0.65	0.0003	93%	< 0.00001	R
Dominant model (GlnGln + ArgGln vs. ArgArg)							
Overall		0.08	0.04–0.18	< 0.00001	92%	< 0.00001	R
Ethnicity	Asian	0.06	0.03–0.12	< 0.00001	86%	< 0.00001	R
	Caucasian	0.28	0.11–0.68	0.005	81%	0.02	R
Degree of cervical lesion	CC	0.07	0.03–0.17	< 0.00001	90%	< 0.00001	R
	CC+CIN	0.11	0.04–0.33	< 0.0001	95%	< 0.00001	R
Recessive model (GlnGln vs. ArgGln + ArgArg)							
Overall		0. 80	0.63–1.01	0.06	63%	0.003	R
Ethnicity	Asian	0.70	0.61–0.81	< 0.00001	0%	0.43	R
	Caucasian	1.14	0.30–4.38	0.85	94%	< 0.0001	R
Degree of cervical lesion	CC	0.90	0.67–1.22	0.50	64%	0.01	R
	CC+CIN	0.79	0.53–1.16	0.22	81%	0.0001	R
Homozygous genetic model (GlnGln vs. ArgArg)							
Overall		0.50	0.33–0.75	0.0009	55%	0.02	R
Ethnicity	Asian	0.44	0.28–0.68	0.0002	42%	0.10	R
	Caucasian	0.77	0.23–2.53	0.67	84%	0.01	R
Degree of cervical lesion	CC	0.56	0.32–0.99	0.05	61%	0.02	R
	CC+CIN	0.68	0.31–1.48	0.33	85%	0.0002	R
Heterozygous genetic model (ArgGln vs. ArgArg)							
Overall		0.57	0.45–0.72	< 0.00001	36%	0.13	F
Ethnicity	Asian	0.54	0.40–0.72	< 0.0001	48%	0.06	F
	Caucasian	0.63	0.41–0.97	0.03	0%	0.59	F
Degree of cervical lesion	CC	0.57	0.37–0.89	0.01	36%	0.15	R
	CC+CIN	0.83	0.48–1.43	0.50	69%	0.02	R

CC: cervical cancer; CIN: cervical intraepithelial neoplasia; F: fixed-effect model; R: random-effect model; OR: odds ratio; 95% CI : 95% confidence interval.

With regard to XRCC1 Arg280His polymorphism, four articles including 2015 objects (784 cases and 1231 controls) offered data about the association between it and cervical cancer risk. On the whole, the heterogeneity among studies were quite huge, the random model was employed to weigh the strength of the association. While the remarkable link between this genetic locus and cervical cancer wasn't witnessed in all models (*P* > 0.05). However, the heterogeneity among studies droped to zero when excluding the study which didn't conform to HWE. Despite of this, the pooled results stayed stable when we eliminated the one [23]. (Table 4)

**Figure 2:**
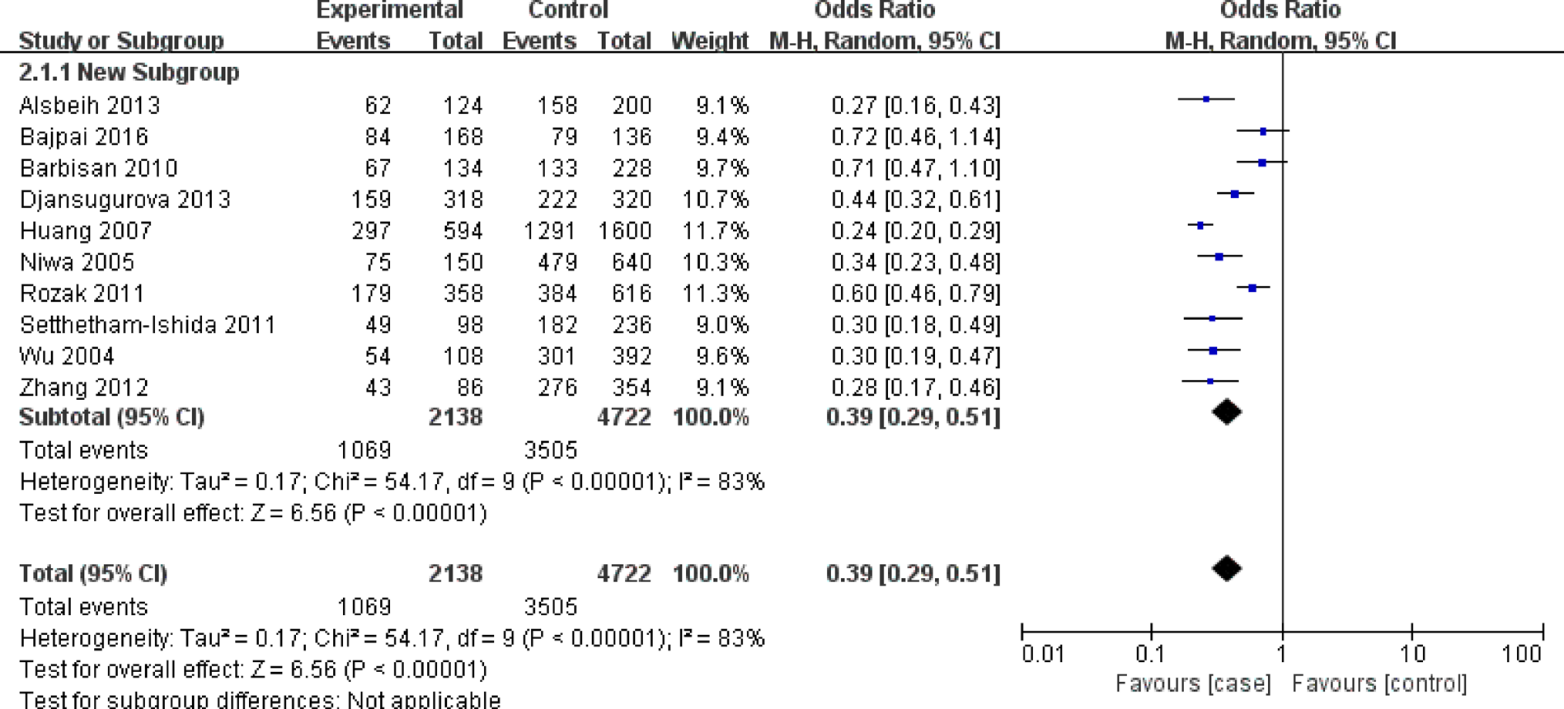
Meta-analysis of the association between XRCC1 Arg399Gln polymorphism and the risk of cervical cancer in allele model CI: confidence interval; OR: odds ratio.

**Figure 3:**
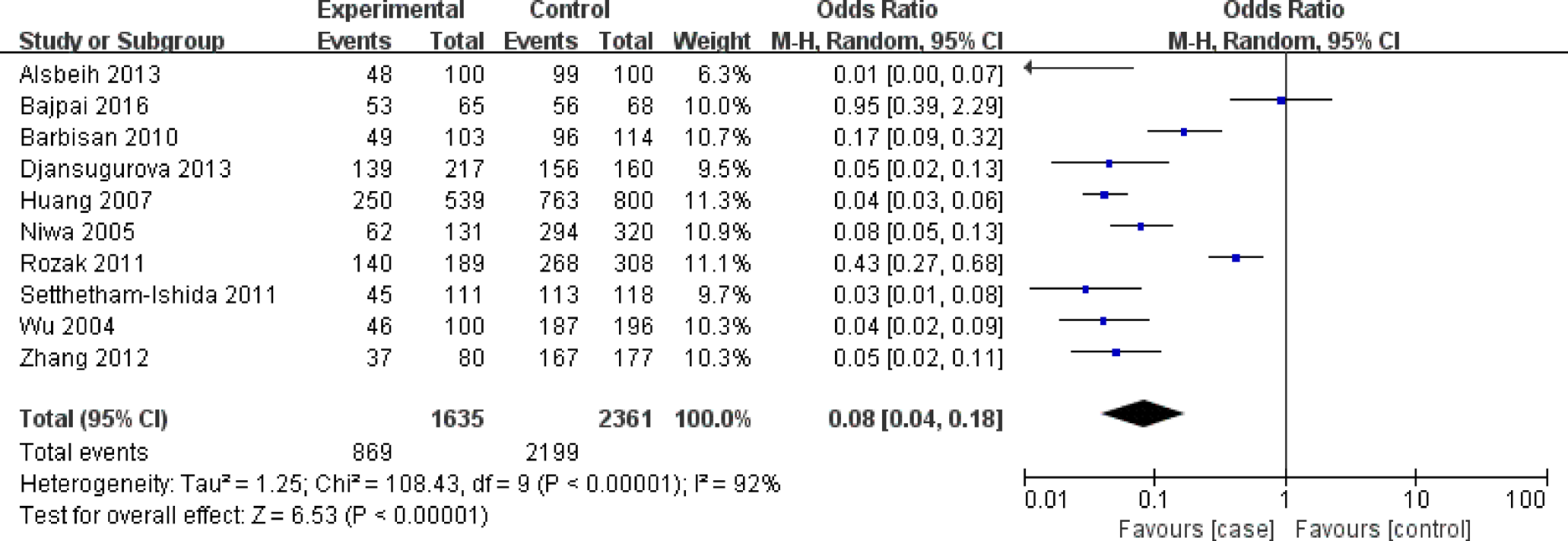
Meta-analysis of the association between XRCC1 Arg399Gln polymorphism and the risk of cervical cancer in dominant model CI: confidence interval; OR: odds ratio.

**Figure 4:**
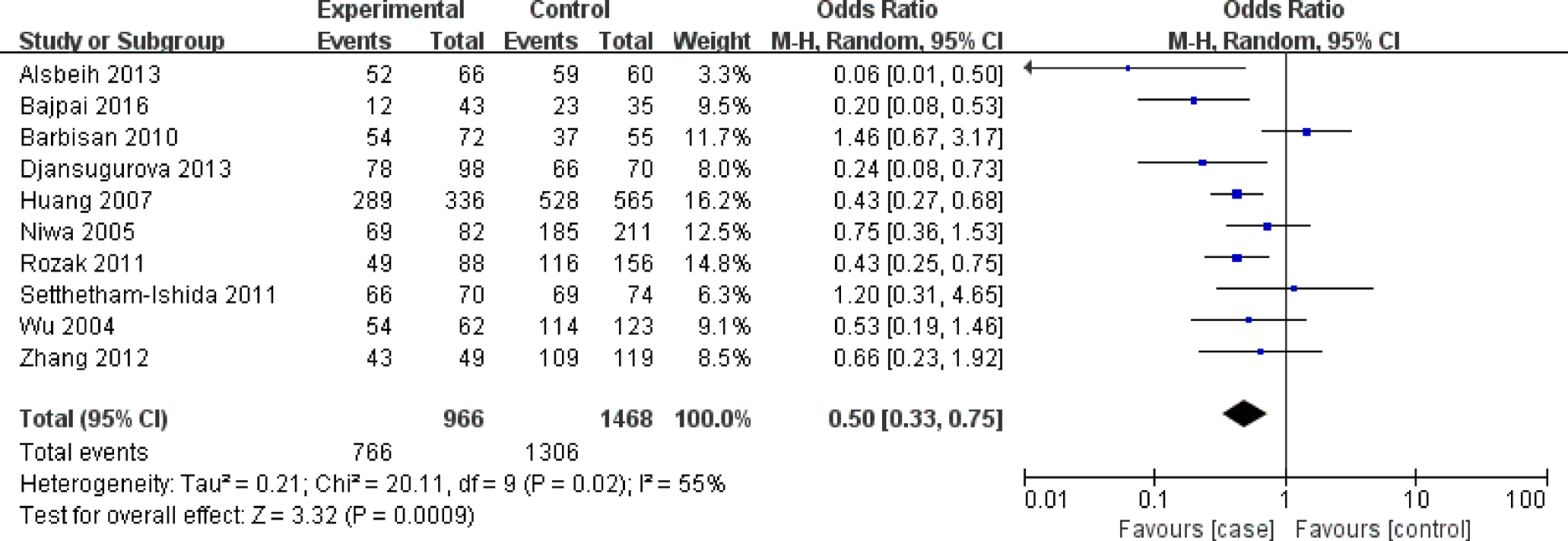
Meta-analysis of the association between XRCC1 Arg399Gln polymorphism and the risk of cervical cancer in homozygous model CI: confidence interval; OR: odds ratio.

**Figure 5:**
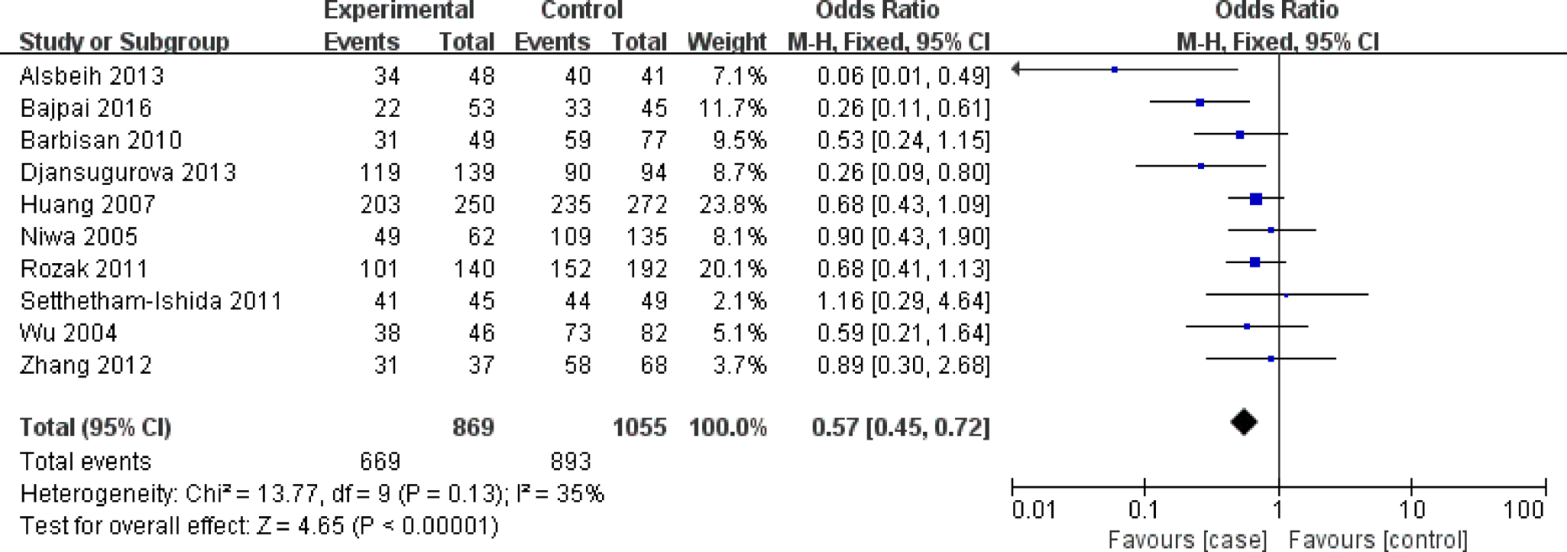
Meta-analysis of the association between XRCC1 Arg399Gln polymorphism and the risk of cervical cancer in heterozygous model CI: confidence interval; OR: odds ratio.

**Figure 6:**
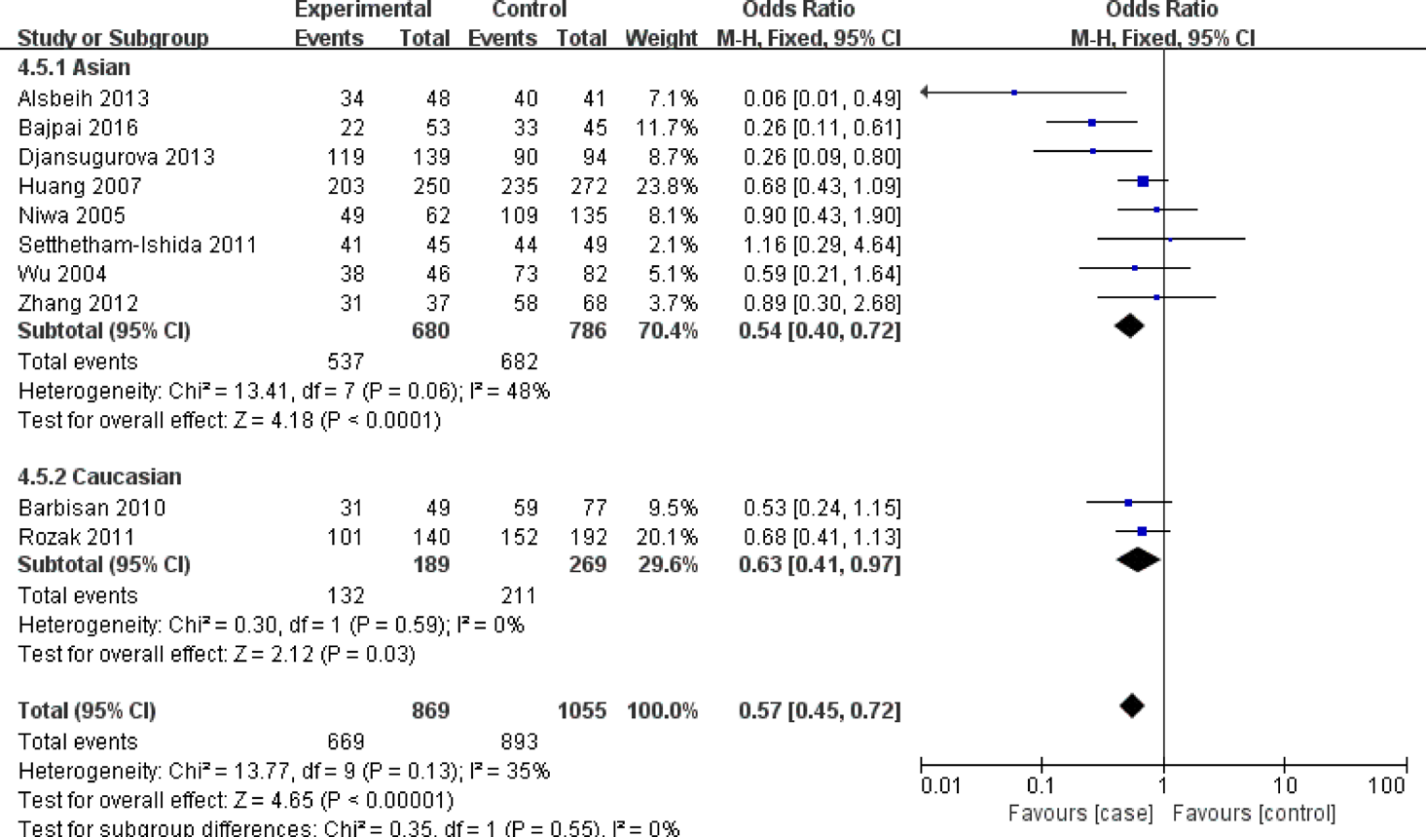
Subgroup analysis of the association between XRCC1 Arg399Gln polymorphism and the risk of cervical cancer stratified by ethnicity in heterozygous model CI: confidence interval; OR: odds ratio.

In terms of XRCC1 Arg399Gln polymorphism, ten studies involving 1635 cancer patients and 2361 controls presented available data about this locus and cervical cancer risk. The Arg399Gln polymorphism decreased cervical cancer susceptibility in four genetic models: allele model (Gln vs. Arg: OR = 0.39, 95% CI = 0.29–0.51, *P* < 0.00001), dominant model (GlnGln + ArgGln vs. ArgArg: OR = 0.08, 95% CI = 0.04–0.18, *P* < 0.00001), homozygous model (GlnGln vs. ArgArg: OR = 0.50, 95% CI = 0.33–0.75, *P* = 0.0009), heterozygous model (ArgGln vs. ArgArg: OR = 0.57, 95% CI = 0.45–0.72, *P* < 0.00001). (Figures 2, 3, 4, 5) While there was no significant difference in recessive model (GlnGln vs. ArgGln + ArgArg: OR = 0.80, 95% CI = 0.63–1.01, *P* = 0.06). All the stuies were in accordance with HWE except one study [24]. The trend of summary ORs remained stable after excluding the one. The subgroup analysis stratified by ethnicity revealed that there still exited obvious association between this polymorphism and decreased cervical cancer among the Asian (Gln vs. Arg: OR = 0.34, 95% CI = 0.26–0.43, *P* = 0.00001; GlnGln + ArgGln vs. ArgArg: OR=0.06, 95% CI = 0.03–0.12, *P* < 0.00001; GlnGln vs. ArgGln + ArgArg: OR = 0.70, 95% CI = 0.61–0.81, *P* < 0.00001; GlnGln vs. ArgArg: OR = 0.44, 95% CI = 0.28–0.68, *P* = 0.002; ArgGln vs. ArgArg: OR = 0.54, 95% CI = 0.40- 0.72, *P* < 0.0001) and the Caucasian (Gln vs. Arg: OR = 0.63, 95% CI = 0.51–0.79, *P* < 0.0001; GlnGln + ArgGln vs. ArgArg: OR = 0.28, 95% CI = 0.11–0.68, *P* = 0.005; ArgGln vs. ArgArg: OR = 0.63, 95% CI = 0.41–0.97, *P* = 0.03). (Figure 6) In the subgroup analysis by the degree of cervical lesion (cervical cancer, cervical cancer + CIN), the Arg399Gln polymorphism reduced the risk of both cervical cancer and CIN. (Table 4)

### Detection for heterogeneity

Considering the great heterogeneity among studies, the random-effect model was applied and subgroup analysis stratified by ethnicity (Figure 6) and the degree of cervical lesion was performed. Nevertheless, the comprehensive results stayed stable. Furthermore, the meta-regression of ethnicity showed no obvious difference (*P* > 0.05), implying that the ethnicity exerted no influence on the association between the XRCC1 Arg399Gln polymorphism and the risk of cervical cancer.

### Sensitivity analysis

**Figure 7:**
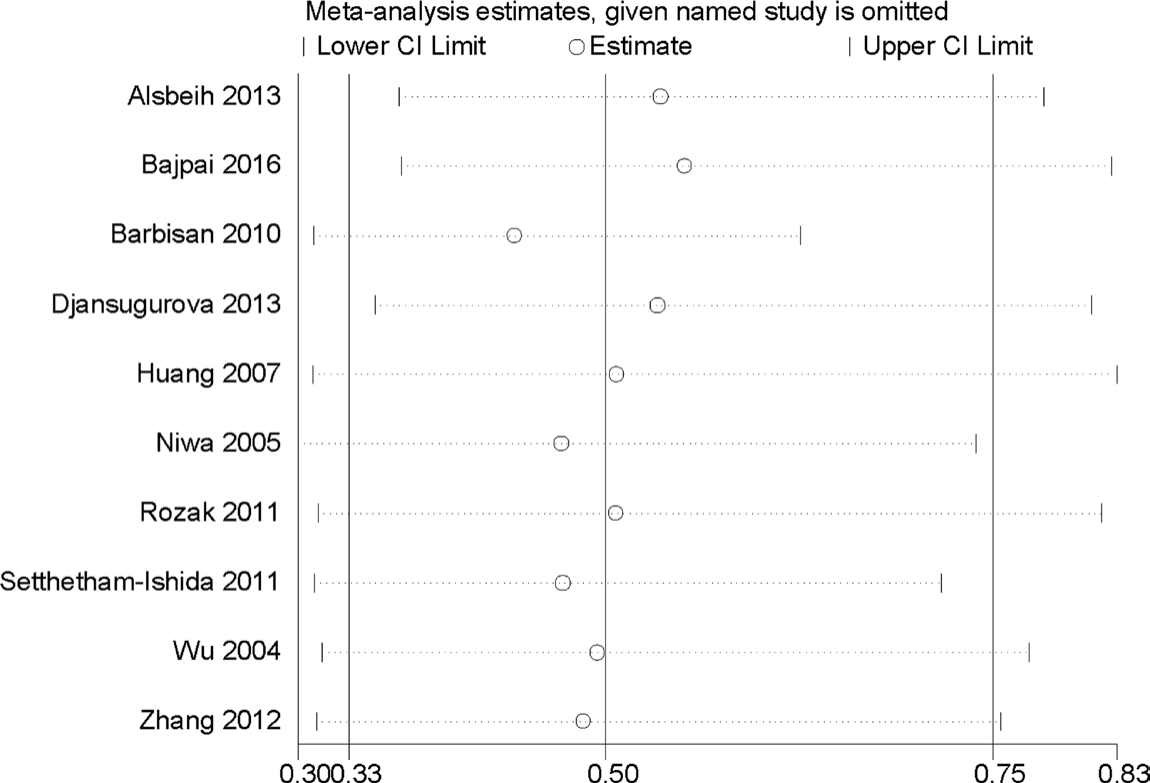
Sensitivity analysis of the association between XRCC1 Arg399Gln polymorphism and the risk of cervical cancer in homozygous model

Although some studies wasn't consistent with the balance of HWE in control groups (*P* < 0.05), yet the final results were not substantially altered after excluding those. Simultaneously, the studies with quite large or small sample sizes were deleted one by one in order to test the stability of pooled results. Moreover, sequential deletion of each study was utilized to perform sensitivity analysis in all models. However, the pooled ORs did not show quantitative changes when excluding any study, suggesting that the results of this meta-analysis were stable and reliable. Sensitivity analysis of the association between the XRCC1 Arg399Gln polymorphism and the risk of cervical cancer in homozygous model was showed Figure 7.

### Publication bias

**Figure 8:**
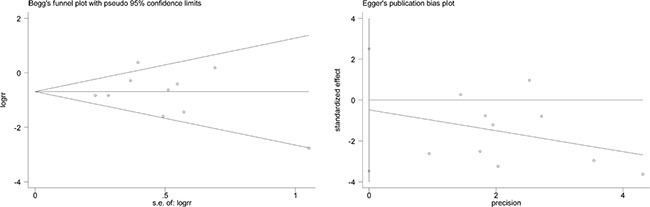
Publication bias of XRCC1 Arg399Gln polymorphism in homozygous model was assessed by Begg's test and Egger's test, suggesting that there was no statistical evidence for publication bias in this meta-analysis (P > 0.05)

The Begg's test and Egger's test were done in all models showing that there was no statistical evidence for publication bias. Publication bias of the XRCC1 Arg399Gln polymorphism in homozygous model was shown in Figure 8 (*P* > 0.05).

## DISCUSSION

Cervical cancer is still the second most common malignant tumor among women and heavily threatens women's health in the world. To improve this embarrassing situation, risk factors concerning cervical cancer should be indentified timely and controlled effectively. There have exited several case-control studies focusing on the relationship between individual susceptibility or genetic variants and cervical cancer [17, 18, 21, 24, 26]. However, the results remained conflicting rather than conclusive. Because a single study may have been underpowered to detect the effect of XRCC1 polymorphisms on cervical cancer risk, yet a quantitative synthesis of accumulative data from all available studies may provide convincing evidence. So a meta-analysis of ten available studies involving 2092 cervical cancer cases and 2803 controls was performed, expecting to derive a more precise estimation of the association between the XRCC1 polymorphism and cervical cancer susceptibility. Our results showed that there was no obvious association between XRCC1 Arg194Trp or Arg280His and cervical cancer susceptibility. Although we did subgroup analysis and sensitivity analysis, the trend of pooled results still remained identical, suggesting that the comprehensive results were quite stable. As to the Arg399Gln polymorphism, it reduced the risk of cervical cancer sharply. Likewise, we performed subgroup analysis and sensitivity analysis, the summary results still hinted a positive relationship between the Arg399Gln polymorphism and the decreased risk of cervical cancer.

Certainly, there have emerged several other meta-analysises concerning the link between XRCC1 polymorphisms and cervical cancer risk. A latest meta-analysis exploring the association between the Arg399Gln polymorphism and cervical cancer showed that the Arg399Gln polymorphism increased the risk of cervical cancer [27]. The result contradicted ours and the reasons may include the following. On the one hand, the number of databases we searched was bigger, resulting in more available studies in English were included. On the another, the quantity of subjects involving in present meta-analysis was greater, which surely strengthened the persuasive power of this research. Another meta-analysis noted that the Arg399Gln polymorphism elevated the risk of cervical cancer in Chinese population [28]. However, the number of included studies was seven, less than the present one. Moreover, it only included the Chinese population, which undoubtedly weakened the strength of the conclusion. Li et al. held that the Arg194Trp polymorphism increased the risk of cervical cancer, while there was no association between the Arg399Gln or Arg280His polymorphism and cervical cancer risk [29]. But the meta-analysis was done five years ago and the number of databases was less than the present, which may explain the discrepancy in the results. Mei et al. showed that the Arg194Trp polymorphism increased the risk of cervical cancer and the Arg399Gln polymorphism elevated the risk of cervical cancer only in Asian population, while there was no association between the Arg280His polymorphism and cervical cancer risk [30]. While the meta-analysis was performed based on only two databases and it included studies without language limits, which may account for the distinction. As you see, the previous meta-analysises either focused on only one polymorphism or only one race or included fewer studies. Yet the present meta-analysis involved all studies of moderate to high quality according to prescribed inclusion and exclusion criteria, so the strength of this study was stronger than those past studies. Simultaneously, even though we performed subgroup analysis and sensitivity analysis, the pooled results still remained stable, supporting that this study was of great credit and persuasiveness.

Likewise, some limitations of this meta-analysis should be mentioned even though considerable effort and resources have been put into testing the possible association between the XRCC1 polymorphism and cervical cancer risk. On the one hand, we retrieved relevant articles only through electronic databases, leading to a potential bias caused by the lack of unpublished articles which would not be available in the electronic databases. On the other, although the great heterogeneity among studies had no effect on the pooled result, yet the heterogeneity could not be neglected completely.

To conclude, the current meta-analysis indicated that the XRCC1 Arg399Gln polymorphism decreased the risk of cervical cancer, while the Arg194Trp and Arg280His polymorphisms were not associated with cervical caner risk. Certainly, to further evaluate the association between XRCC1 polymorphisms and cervical cancer susceptibility, a well-designed large-scale multicenter study is warranted to confirm the finding.

## MATERIALS AND METHODS

### Literature searching strategy

A systematic literature search was done through PubMed, Web of Science, EMBASE and the Cochrane Library up to July 2016 in English. The search terms included “X-ray repair cross complementing protein 1”, “XRCC1”, “Arg194Trp”, “rs1799782”, “Arg280His”, “rs25489”, “Arg399Gln” or “rs25487”; “poly-morphism”, “variant”, “genotype”, “polymorphism” or “SNP”; “cervical” or “cervix”; “cancer”, “carcinoma”, “neoplasm”, “tumor” or “ malignancy “and the combinations. Besides, the relevant references of identified studies were screened carefully for potential articles.

### Inclusion and exclusion criteria

The included studies have to meet the following criteria: 1) investigating the association between XRCC1 polymorphisms (Arg194Trp, Arg280His and Arg399Gln) and risk of cervical cancer; 2) studies on human beings; 3) genotype frequencies were available both in case and control groups; 4) subjects in control groups should have no cancer history, previous radiotherapy and chemotherapy history and a family history of tumor; 5) the diagnosis of the cases was based on pathology. The study with the following criteria was excluded: 1) abstracts, case reports, letters, comments, editorials, reviews and mata-analysises; 2) studies lacking relevant data. What's more, the most recent study was included once the studies were duplicated. Any one study was screened by two authors independently and disagreements were resolved by discussing with a third author.

### Data extraction and synthesis

Two investigators simutaneously extracted characteristics of the included studies according to the inclusion and exclusion criteria and the results were checked by a third reviewer. The data extracted from each study included first author, year of publication, country of origin, ethnicity, source of the control group, genotyping method and numbers of case and control subjects. Ethnicity was classified as ‘‘Caucasian’’, ‘‘Asian’’ and ‘‘Mixed’’.

### Quality assessment

The methodological quality assessment was performed based on the modified scoring system used for studies in genetic epidemiological issues. [31] Points were awarded on the basis of representativeness of cases, source of controls, HWE in controls, genotyping examination and association assessment. Total score ranged from 0 (lowest quality) to 8 (highest quality). A study with a score of 6 or higher was classified as high quality and vice versa.

### Statistical analysis

Review Manage version 5.2.0 (The Cochrane Collaboration, 2012) and STATA version 11.0 software (StataCorp LP, College Station, TX) were applied to carry out statistical analysis. The association between XRCC1 polymorphisms and cervical cancer risk was estimated in the allele model, the dominant model, the recessive model, the homozygous genetic model and the heterozygous genetic model. To evaluate the strength of associations, the summary odds ratio (OR) and 95% confidence interval (CI) were calculated through fixed/random effects mode. *P* < 0.05 was considered statistically significant. To test the heterogeneity among studies, we assumed the *I*^2^ and *Q* statistic. We adopted random effect model if there was great heterogeneity (*I*^2^ greater than 50%). Otherwise, we adopted the fixed effect model. At the same time, we conducted a subgroup analysis according to ethnicities. To assess the stability of the finding, we performed sensitivity analysis. Each study involved in this meta-analysis was deleted respectively to reflect the influence of the individual data exerted on the pooled OR. HWE of the genotype frequencies in the control group of each study was assessed by χ^2^ test and *P* > 0.05 was considered to be consistent with HWE [32]. For the studies which did not live up to HWE, we reassessed the association by eliminating them. The Begg's funnel plot and Egger's test were used to evaluate the possibly exiting publication bias [33, 34].

## References

[1] Shi YH, Wang B, Xu BP, Jiang DN, Zhao DM, Ji MR, Zhou L, Li X, Lu CZ (2016). The association of six non-synonymous variants in three DNA repair genes with hepatocellular carcinoma risk: a meta-analysis. J Cell Mol Med.

[2] Sanjari Moghaddam A, Nazarzadeh M, Bidel Z, Karamatinia A, Darvish H, Mosavi-Jarrahi A (2016). XRCC1 Gene Polymorphisms and Breast Cancer Risk: A Systematic Review and Meta- Analysis Study. Asian Pac J Cancer Prev.

[3] Qi L, Yu HQ, Zhang Y, Ding LJ, Zhao DH, Lv P, Wang WY, Xu Y (2016). A Comprehensive Meta-analysis of Genetic Associations Between Key Polymorphic Loci in DNA Repair Genes and Glioma Risk. Mol Neurobiol.

[4] Li Y, Bai O, Cui J, Li W (2016). Genetic polymorphisms in the DNA repair gene, XRCC1 associate with non-Hodgkin lymphoma susceptibility: A systematic review and meta-analysis. Eur J Med Genet.

[5] Li F, Wang J, Chen M (2016). Single nucleotide polymorphisms in DNA repair genes and the risk of laryngeal cancer: A meta-analysis. Biomed Pharmacother.

[6] Yuanyuan M, Xiaoming Y, Lijie Z, Ninghan F (2015). Association between codon 399 polymorphism in the X-ray repair cross-complementing group 1 gene and risk of prostate cancer in Asians: A study of 4,479 cases and 4,281 controls. Pak J Med Sci.

[7] Yuan Z, Li J, Hu R, Jiao Y, Han Y, Weng Q (2015). Predictive assessment in pharmacogenetics of XRCC1 gene on clinical outcomes of advanced lung cancer patients treated with platinum-based chemotherapy. Sci Rep.

[8] Wang F, Zhao Q, He HR, Zhai YJ, Lu J, Hu HB, Zhou JS, Yang YH, Li YJ (2015). The association between XRCC1 Arg399Gln polymorphism and risk of leukemia in different populations: a meta-analysis of case-control studies. Onco Targets Ther.

[9] Huang J, Zhang J, Zhao Y, Liao B, Liu J, Li L, Liao M, Wang L (2011). The Arg194Trp polymorphism in the XRCC1 gene and cancer risk in Chinese Mainland population: a meta-analysis. Mol Biol Rep.

[10] Goode EL, Ulrich CM, Potter JD (2002). Polymorphisms in DNA repair genes and associations with cancer risk. Cancer Epidemiol Biomarkers Prev.

[11] Caldecott KW, Tucker JD, Stanker LH, Thompson LH (1995). Characterization of the XRCC1-DNA ligase III complex in vitro and its absence from mutant hamster cells. Nucleic Acids Res.

[12] Dianov GL, Prasad R, Wilson SH, Bohr VA (1999). Role of DNA polymerase beta in the excision step of long patch mammalian base excision repair. J Biol Chem.

[13] Thompson LH, West MG (2000). XRCC1 keeps DNA from getting stranded. Mutat Res.

[14] Zhang L, Ruan Z, Hong Q, Gong X, Hu Z, Huang Y, Xu A (2012). Single nucleotide polymorphisms in DNA repair genes and risk of cervical cancer: A case-control study. Oncol Lett.

[15] Wu MT, Liu CL, Ho CK, Wu TN (2004). Genetic polymorphism of p53 and XRCC1 in cervical intraepithelial neoplasm in Taiwanese women. J Formos Med Assoc.

[16] Wang SS, Bratti MC, Rodriguez AC, Herrero R, Burk RD, Porras C, Gonzalez P, Sherman ME, Wacholder S, Lan ZE, Schiffman M, Chanock SJ, Hildesheim A (2009). Common variants in immune and DNA repair genes and risk for human papillomavirus persistence and progression to cervical cancer. J Infect Dis.

[17] Settheetham-Ishida W, Yuenyao P, Natphopsuk S, Settheetham D, Ishida T (2011). Genetic risk of DNA repair gene polymorphisms (XRCC1 and XRCC3) for high risk human papillomavirus negative cervical cancer in Northeast Thailand. Asian Pac J Cancer Prev.

[18] Roszak A, Lianeri M, Jagodzinski PP (2011). Involvement of the XRCC1 Arg399Gln gene polymorphism in the development of cervical carcinoma. Int J Biol Markers.

[19] Niwa Y, Matsuo K, Ito H, Hirose K, Tajima K, Nakanishi T, Nawa A, Kuzuya K, Tamakoshi A, Hamajima N (2005). Association of XRCC1 Arg399Gln and OGG1 Ser326Cys polymorphisms with the risk of cervical cancer in Japanese subjects. Gynecol Oncol.

[20] Huang J, Ye F, Chen H, Lu W, Xie X (2007). The nonsynonymous single nucleotide polymorphisms of DNA repair gene XRCC1 and susceptibility to the development of cervical carcinoma and high-risk human papillomavirus infection. Int J Gynecol Cancer.

[21] Djansugurova LB, Perfilyeva AV, Zhunusova GS, Djantaeva KB, Iksan OA, Khussainova EM (2013). The determination of genetic markers of age-related cancer pathologies in populations from Kazakhstan. Front Genet.

[22] Barbisan G, Perez LO, Difranza L, Fernandez CJ, Ciancio NE, Golijow CD (2011). XRCC1 Arg399Gln polymorphism and risk for cervical cancer development in Argentine women. Eur J Gynaecol Oncol.

[23] Bajpai D, Banerjee A, Pathak S, Thakur B, Jain SK, Singh N (2016). Single nucleotide polymorphisms in the DNA repair genes in HPV-positive cervical cancer. Eur J Cancer Prev.

[24] Alsbeih G, Al-Harbi N, El-Sebaie M, Al-Badawi I (2013). HPV prevalence and genetic predisposition to cervical cancer in Saudi Arabia. Infect Agent Cancer.

[25] Wu MT, Chen SY, Wu TN, Hwang HY, Ho CK, Lee LH, Wu SC (2003). No association between polymorphisms of the DNA repair geneXRCC1 and cervical neoplasm risk. Environ Health Prev Med.

[26] Zhang X, Zhang L, Tian C, Yang L, Wang Z (2014). Genetic variants and risk of cervical cancer: epidemiological evidence, meta-analysis and research review. BJOG.

[27] Liu DY, Liang HC, Xiao XM (2015). Association between the XRCC1 Arg399Gln polymorphism and risk of cervical carcinoma: a meta-analysis. Genet Mol Res.

[28] Zhang F, Li B, Wu HY, Shang LX (2016). Association between X-Ray Repair Cross-Complementing Group 1 Arg399Gln Polymorphism and Cervical Cancer Risk: A Meta-Analysis in the Chinese Population. Gynecol Obstet Invest.

[29] Li Y, Liu F, Tan SQ, Wang Y, Li SW (2012). X-ray repair cross-complementing group 1 (XRCC1) genetic polymorphisms and cervical cancer risk: a huge systematic review and meta-analysis. PLoS One.

[30] Mei J, Duan HX, Wang LL, Yang S, Lu JQ, Shi TY, Zhao Y (2014). XRCC1 polymorphisms and cervical cancer risk: an updated meta-analysis. Tumour Biol.

[31] Niu YM, Du XY, Cai HX, Zhang C, Yuan RX, Zeng XT, Luo J (2015). Increased risks between Interleukin-10 gene polymorphisms and haplotype and head and neck cancer: a meta-analysis. Sci Rep.

[32] Mantel N, Haenszel W (1959). Statistical aspects of the analysis of data from retrospective studies of disease. J Natl Cancer Inst.

[33] Egger M, Davey Smith G, Schneider M, Minder C (1997). Bias in meta-analysis detected by a simple, graphical test. BMJ.

[34] Begg CB, Mazumdar M (1994). Operating characteristics of a rank correlation test for publication bias. Biometrics.

